# Pediatric Hospitalizations for Asthma: Use of a Linked File to Separate Person-level Risk and Readmission

**Published:** 2004-03-15

**Authors:** Jonathan C. Wallace, Charles E. Denk, Lakota K. Kruse

**Affiliations:** Maternal and Child Health Epidemiology Program, New Jersey Department of Health and Senior Services; Maternal and Child Health Epidemiology Program, New Jersey Department of Health and Senior Services, Trenton, NJ; Maternal and Child Health Epidemiology Program, New Jersey Department of Health and Senior Services, Trenton, NJ

## Abstract

**Introduction:**

Disparities in asthma hospitalization by gender, age, and race/ethnicity are thought to be driven by a combination of 2 factors: disease severity and inadequate health care. Hospitalization data that fail to differentiate between numbers of admissions and numbers of individuals limit the ability to derive accurate conclusions about disparities and risks.

**Methods:**

Hospitalization records for pediatric asthma patients (aged one to 14 years) were extracted from New Jersey Hospital Discharge Files (for the years 1994 through 2000) and then linked by patient identifiers using a probabilistic matching algorithm. The analysis file contained 30,400 hospital admissions for 21,016 children. Hospitalization statistics were decomposed into persons hospitalized and number of hospitalizations. Analysis of readmission within 180 days of discharge used additional records from 2001 to avoid bias due to truncated observation.

**Results:**

Overall, 22.9% of children in our analysis had repeat asthma admissions within the same age interval, accounting for 30.9% of all hospitalizations. Also among all children, 11.7% had at least one readmission within 180 days of a prior discharge. The risk of hospitalization was higher for boys, decreased by age for both genders, was lowest for white children and highest for black children. Readmission rates were higher for black and Hispanic girls than boys in older age groups, but were otherwise relatively uniform by gender and age.

**Conclusion:**

Decomposition of ratios of total hospitalizations to population illuminates components of risk and suggests specific causes of disparity.

## Introduction

Asthma is one of the most common chronic conditions in the United States and is often cited as the most frequent reason for preventable hospital admissions among children (1-4). The United States Department of Health and Human Services' *Healthy People 2010: Objectives for Improving Health* established a goal to "reduce asthma morbidity, as measured by a reduction in hospitalizations" (5).

The Asthma and Allergy Foundation of America estimates that there are 142,000 children with asthma in New Jersey (6). Hospital discharge data from 1985 through 2000 indicate that asthma is a major cause of hospitalization for all ages in New Jersey, accounting for approximately 1% of New Jersey's average 1.4 million hospital discharges each year (7). New Jersey mirrors national disparities in asthma hospitalization rates among various population groups by age, gender, and race (1,2).

Few state asthma surveillance systems to date differentiate between the number of individuals hospitalized and the number of admissions, the latter of which can be numerous during an individual's lifetime. The difference between the number of individuals hospitalized and the number of admissions can lead to several forms of bias. First, total hospital admissions overstate the number of individuals affected by severe asthma. Second, the repeat admissions of some individuals may obscure the true sociodemographic distribution of severe disease. Third, routine inference from hospitalizations to individuals implicitly assumes that patterns of readmission reinforce sociodemographic differences in person-level risk or are neutral. Hospitalization for asthma implies more severe disease, less adequate preventive care, or both. Failure to distinguish persons from hospitalizations undermines inferences about the contribution of either of these 2 classes of causation.

The objectives of this study were to use linked hospital asthma admission data for children to accomplish the following: 1) deduplicate records of asthma hospitalizations and assess the scope of readmissions; 2) investigate relative risks of ever being hospitalized; and 3) examine the frequency of hospitalization for each individual admitted and assess the risk of readmission for asthma within 180 days of a prior discharge.

## Methods

The study population was defined as all asthma hospitalizations experienced by New Jersey resident children aged one to 14 years from 1994 through 2000. Records from New Jersey Hospital Discharge Files (UB-92) were linked to identify patients with multiple asthma hospitalizations. Asthma hospitalizations were defined using the Centers for Disease Control and Prevention case definition: a primary diagnosis of ICD-9 Code 493 (8). AutoMatch, a probabilistic matching program, was used to link patients with more than one hospitalization in the data subset (9,10). Variables used in the linking process included the following: last name, first name, date of birth, municipality, zip code, race/Hispanic origin, hospital, medical record number, and insurer identification number. In probabilistic matching for deduplication, pools of candidate matches are identified by one or more variables such as last name and birth date. A second set of variables is used to score the similarity of each candidate pair, and the highest scored candidates are linked subject to a minimum cutoff score. The linkage process is iterative, so that variables used to identify candidate matches at one stage were used for confirmatory scoring in other stages and vice versa.

Hospitalization records identified the patient's age, gender, and race/ethnicity. Patient ages were grouped into the following age categories at each hospital admission: one to 4 years, 5 to 9 years, and 10 to 14 years. Children younger than one year were excluded because of issues of uncertainty among physicians surrounding the diagnosis and coding of asthma for children at this age. After age and calendar time exclusions, 32,825 hospitalization records representing 22,990 individuals were available. However, 399 records (1.2%) had missing data for race/ethnicity, and 615 additional persons (3.1%) had inconsistent data across multiple records. In both cases, all records for each person were coded using the following hierarchy:

- If any records indicated race as Hispanic, then the person was coded "Hispanic" for all hospitalizations.
- If any records indicated race as black, then the person was coded "black" for all hospitalizations.
- If no records contradicted race as white, then the person was coded "white" for all hospitalizations.

The final data extract had 30,400 hospitalization records representing 21,016 white, black, and Hispanic persons. Asian and other non-Hispanic individuals were not included in this analysis because of the relatively small number of hospitalizations.

For our analysis, *hospitalization ratios* are defined as the number of hospitalizations in a population subgroup, including multiple readmissions of the same patient. They were calculated using data on age, gender, and ethnic origin from the 2000 Census as the denominator. Intercensal estimates would not enhance the analysis. Because 7 years of hospitalization records were extracted, census denominators were multiplied by 7.

*Person-level hospitalization rates* are defined as the number of distinct individuals hospitalized within a population subgroup (including age groups), and used the same 2000 Census denominators (age, gender, and ethnic origin) as hospitalization ratios. Records shared a common person identifier within the linked file. We were thus able to count the number of unique individuals hospitalized within any population subgroup. Individuals who were hospitalized at ages in different categories were counted once per age group. The *frequency of admission per child* is the ratio of total admissions within an age group to the number of individual children in the age group — essentially, the average number of admissions per individual child.

For a more precise analysis of readmissions, we treated each admission as a prospective opportunity for readmission and checked for a succeeding admission for that individual within 180 days from the date of discharge. We also accessed records from the first half of 2001, so that loss to follow-up would not occur for admissions late in 2000. Age group was classified by the *earlier admission in each potential pair*, and the entire 180-day readmission window was used even if the child aged out of his or her original age group. *Readmission rates* are the proportion of admissions in each subpopulation with a succeeding readmission in the time window. Although it departs from the logic of decomposing hospitalization ratios, the analysis of readmission events over a fixed period, constructed to minimize censoring bias, is a stronger approach with fewer inherent limitations.

We judged that the period of 180 days was an appropriate window for clinical follow-up of a chronic condition compared, for example, to much shorter times required for complications from surgical procedures. Also, since aggregate rates of asthma hospitalization have a strong annual cycle, we wanted an interval short enough to avoid confounding with that periodicity. Analysis using other time intervals did not yield substantially different results.

All analysis was performed using SAS Release 8.01 (11). Since the data set captures virtually all persons and hospitalizations for the period examined, statistical inferences based on sampling variability are not applicable. Systematic errors of omission, classification, linkage, and other biases do not follow the same statistical laws as sampling errors. The practical significance of subgroup differences, and whether they exceed expectations of error, must be judged on external rather than statistical criteria.

## Results

From 1994 through 2000, 21,016 New Jersey children accounted for 30,400 asthma hospitalizations in New Jersey (Table 1). Overall, 4808 children (22.9% of all children hospitalized for asthma in New Jersey) experienced multiple admissions; their 9384 duplicate admissions accounted for 30.9% of all pediatric asthma admissions. Within this group, 2459 children (11.7% of all children hospitalized for asthma in New Jersey) experienced at least one readmission within 180 days of a prior asthma discharge, totaling 4340 admissions. These latter readmissions, however, accounted for 14.3% of all childhood/pediatric hospitalizations for asthma in New Jersey.

**Figure 1:**
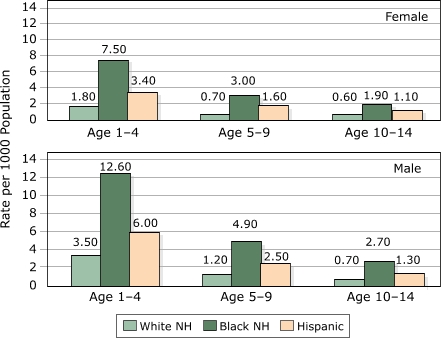
Person-level hospitalization rates for asthma by age, gender, race/ethnicity, New Jersey, 1994–2000. NH indicates non-Hispanic.

**Figure 2:**
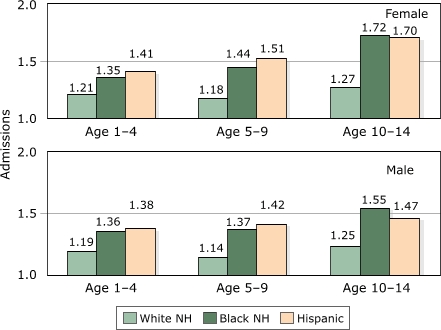
Average admissions per child hospitalized for asthma by age, gender, and race/ethnicity, New Jersey, 1994–2000. NH indicates non-Hispanic.

**Figure 3:**
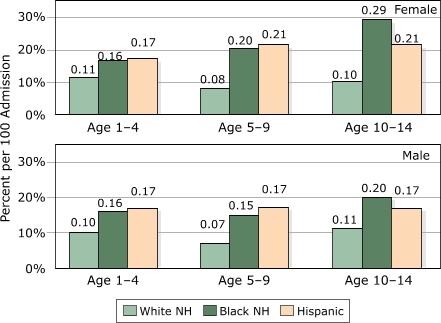
Readmission rates within 180 days of prior asthma discharge by age, gender, and race/ethnicity, New Jersey, 1994–2000. NH indicates non-Hispanic.

Table 2 presents the decomposition of hospitalization ratios into components by gender, age, and race/ethnicity. To illuminate the most salient points of Table 2, we constructed Figures 1-3.

Figure 1 presents person-level hospitalization rates for all individual children (per thousand population) hospitalized for asthma in New Jersey by age, sex and race/ethnicity. Rates are generally higher for boys than girls. For example, rates for boys are approximately twice the rates for girls among white children aged one to 4 years; the difference varies somewhat by age and race/ethnicity. Rates decline with age, especially between the ages of one to 4 years and 5 to 9 years. For example, rates for white girls drop from 1.8 to 0.7 per 1000 between ages one to 4 years and ages 5 to 9 years, and rates for white boys drop from 3.5 to 1.2 per 1,000 for the same age groups. White children experience the lowest rates, black children the highest, and rates for Hispanic children rates fall in between. Generally, black children have about 4 times the risk of hospitalization compared to white children of comparable age and gender; Hispanic children have about twice the risk compared to their white peers.

Figure 2 presents the frequency of admissions per child who has ever been hospitalized for asthma in New Jersey by age, gender, and race/ethnicity. The qualitative differences in relationships compared to the previous figure are striking; in contrast to person-level hospitalization rates, the frequency of admissions is roughly uniform by age for white children and increases by age for black and Hispanic children. Admission frequencies vary for white girls from 1.21 (ages one to 4 years) to 1.18 (ages 5 to 9 years) to 1.27 (ages 10 to 14 years), but for black girls, admission frequencies increase from 1.35 to 1.44 to 1.72 for the same age groups. Generally, the frequency of admission for boys and girls is equal for white children, although girls have higher averages than boys among older blacks and Hispanics. For example, among white children aged one to 4 years ever hospitalized for asthma, girls experience an average of 1.21 admissions while boys have an average of 1.19; the same comparison for Hispanic children ages 10 to 14 years is 1.70 (girls) vs 1.47 (boys). Finally, compared to the relative advantage of Hispanics over blacks in Figure 1, that trend is eliminated or reversed for frequency of admission, depending on the age group.

Figure 3 presents our analysis of readmission rates more precisely calculated as readmission within 180 days of prior discharge and displays the same general patterns as the admission frequencies in Figure 2 with respect to gender, age and race/ethnicity. Again, readmission rates are roughly uniform by age for white children and increase by age for black and Hispanic girls only. Readmission rates vary for white girls from 11% (ages one to 4 years) to 8% (ages 5 to 9 years) and 10% (ages 10 to 14 years), but for black girls they increase from 16% to 20% to 29% (same age groups). Generally, readmission rates for boys and girls are equal for white children, although girls have higher rates than boys among older black and Hispanic children. For example, among white children ages one to 4 years ever hospitalized for asthma, girls experience a readmission rate of 11% while boys have an average of 10%; the same comparison for Hispanic children ages 10 to 14 years is 21% (girls) vs 17% (boys). Black girls ages 10 to 14 years have a very distinctive spike compared to boys and to Hispanic girls of the same age.

To underscore the comparisons discussed above, Table 3 transforms the measures in Table 2 into relative risks and similar ratios. The first 4 columns contrast younger age groups against the 10-to-14 years age group. These columns most clearly support our generalizations and distinctions about age and race/ethnicity. Hospitalization ratios and person-level rates decline dramatically with age across all race/ethnic groups, with slightly larger declines for boys. Admission frequencies and readmission rates have much weaker and usually opposite effects. It is important to note that wherever age has opposite effects on person-level admission and readmission, the overall hospitalization ratio will be smaller than the person-level admission rate. The last 4 columns of Table 3 contrast black and Hispanic children against whites. In general, both person-level hospitalization and readmission components are substantial and in the same direction, which produces even larger relative differentials in hospitalization ratios. The difference between black and Hispanic children is largely a function of person-level hospitalization — every other ratio in these columns is very similar between the two.

## Discussion

Hospitalization ratios in New Jersey have associations with gender, age, and race/ethnicity that are comparable to other recent reports (12-17). Repeat hospitalizations for asthma are a common event for New Jersey children, accounting for almost one third of all admissions for asthma. Much epidemiological evidence and clinical experience suggests that hospitalization for asthma is a mixture of biological disease factors and failures of preventive care. We believe that the significant qualitative differences between person-level hospitalization rates and readmissions provide important leverage to distinguish the influences of the two.

Person-level hospitalization rates give a more precise accounting of how the burden of severe asthma is distributed across individuals (rather than the burden of hospitalization as a distinct event). This is the main implication of the differences between hospitalization ratios and person-level rates in Table 3. On the other hand, readmissions are more than residual events to be eliminated in deduplication. Readmission rates are arguably more driven by issues of disease management, since children in that analysis are more homogeneous — they all have a degree of disease severe enough to require hospitalization. The 2 rates in combination — person-level and readmission — tell the same story as hospitalization ratios, but in a more focused and coherent way.

In general, our findings on readmission rates fit with very simple hypotheses about barriers to appropriate preventive care. Based solely on socioeconomic barriers to access, we would expect comparatively little variation by gender and age (of children, not adults), and we would expect black and Hispanic children to be similar to each other. These expectations are generally met by our data. Person-level rates clearly are more complex, and it would be helpful if we could assess the relative contribution of access and other factors.

For example, numerous studies have documented age and race/ethnic disparities in asthma hospitalization (12-18). Variations in the accessibility and quality of preventive asthma care have been linked to race/ethnicity (19-22). A recent study that explicitly addresses financial access still finds disparities in processes of asthma care such as the use of controller medications (23). Risk factors for asthma morbidity — such as outdoor environment, family smoking, and physical activity — have been well demonstrated to vary by racial and ethnic groups. Less is known about potential biologic factors such as genetic, physiological, pharmacogenomic, and/or environmental-genetic interactions.

In New Jersey, person-level hospitalization rates for black children are 3.2 to 4.3 times higher than for white children (stratified by age and gender, Table 3). This ratio is about twice the disparity in person-level rates experienced by Hispanic children and also twice the disparity in readmission rates for both black and Hispanic children. On the basis of this outsized differential, we observe, as others have, that there could be a biological difference in disease etiology for black children (24,25). Regardless of the cause(s), this disparity demands further investigation.

Numerous studies have documented higher hospitalization ratios for preschool-age children (12-16). In New Jersey, children ages one to 4 years have threefold higher person-level hospitalization rates than preadolescents among girls and more than fourfold higher rates among boys. This differential does not exist at all for readmission rates, contrary to To's findings from Canada (12). This would seem to undermine the hypothesis that preschool-age children present special challenges for home management and lends more support to the distinctive nature of early onset asthma (26,27). On the other hand, there is an anomalous spike in readmission rates for black girls ages 10 to 14 years, which is not mirrored in person-level rates or in black boys or Hispanic girls of the same age. It is more likely that this anomaly is related to asthma management and use of controller medications (28).

Our analysis of linked hospitalizations does not allow us, unfortunately, to explore some issues typical in a cohort analysis. For example, we cannot describe age-specific onset or population prevalence of severe asthma. Since we cannot in most cases identify the first hospitalization for each child, we can only generalize about readmissions by assuming independence among the intervals between hospitalizations.

Matching of annual files does not lend itself, in this case, to the construction and analysis of age cohorts of children. The optimal timeframe for a longitudinal file is limited by the comparability of files over many years and a natural decay in expected matching accuracy. True cohort data, with complete histories from both inpatient and outpatient management, would obviously be much more powerful.

State-level asthma hospitalization surveillance systems like New Jersey's address 2 objectives, both incompletely. Trends in asthma hospitalizations can inform us about prevalence and distribution of the severest forms of the disease. Surveillance of asthma readmissions especially informs us about the effectiveness of asthma care — accessibility of care, management of environmental triggers, and appropriate preventive asthma management. For both objectives, racial and ethnic disparities and age-specific differences are critically important.

## Figures and Tables

**Table 1:** Hospitalization Records for Pediatric Asthma Admissions, New Jersey, 1994–2000

	**Admissions**		**Children**		**Admissions per Child**

	**n**	**%**	**n**	**%**	
All pediatric asthma admissions	30,400	100.0	21,016	100.0	1.45
Repeat asthma admissions within same age interval	9,384	30.9	4,808	22.9	2.95
Readmissions within 180 days of previous discharge^a^	4,340	14.3	2,459	11.7	2.76

a Analysis of readmissions within 180 days of discharge used additional records from 2001 to avoid censoring bias.

**Table 2:** Pediatric Asthma Hospitalizations and Readmissions by Race/Ethnicity, Age, and Gender, New Jersey, 1994–2000

**Race/Ethnicity**	**Age (years)**	**Pediatric Asthma Admissions (n)**	**Children Admitted for Asthma (n)**	**2000 Census (n)**	**Hospitalization Ratio^a^**	**Person-level Hospitalization Rate^b^**	**Mean Hospitalizations per Child Ever Hospitalized for Asthma^c^**	**Readmission Within 180 Days of Prior Discharge^d^(%)**
**Female**								

**White**	1-4	2006	1655	127,941	2.2	1.8	1.21	11
	5-9	1028	871	174,845	0.8	0.7	1.18	8
	10-14	921	726	174,529	0.8	0.6	1.27	10
**Black**	1-4	2353	1746	33,352	10.1	7.5	1.35	16
	5-9	1447	1002	47,256	4.4	3.0	1.44	20
	10-14	1071	621	46,114	3.3	1.9	1.72	29
**Hispanic**	1-4	1283	909	38,335	4.8	3.4	1.41	17
	5-9	795	526	47,347	2.4	1.6	1.51	21
	10-14	565	333	44,174	1.8	1.1	1.70	21

**Male**								

**White**	1-4	3914	3283	134,718	4.2	3.5	1.19	10
	5-9	1782	1570	183,919	1.4	1.2	1.14	7
	10-14	1191	953	184,884	0.9	0.7	1.25	11
**Black**	1-4	4160	3050	34,456	17.2	12.6	1.36	16
	5-9	2311	1688	49,335	6.7	4.9	1.37	15
	10-14	1385	894	47,466	4.2	2.7	1.55	20
**Hispanic**	1-4	2339	1691	39,977	8.4	6.0	1.38	17
	5-9	1257	886	49,711	3.6	2.5	1.42	17
	10-14	592	403	45,770	1.8	1.3	1.47	17

**Total**		30,400	21,016	1,504,129	N/A	N/A	N/A	N/A

**Average**		N/A	N/A	N/A	N/A	2.0	1.45	15

a Per 1000 population. Hospital ratios are defined as the number of pediatric asthma admissions in a population subgroup, including multiple readmissions of the same patient, divided by the number of individuals in a population subgroup, as determined by the 2000 Census, multiplied by 7.

b Per 1000 population. Person-level hospitalization rates are defined as the number of pediatric asthma individuals hospitalized within a population subgroup, divided by the number of individuals in a population subgroup, as determined by the 2000 Census, multiplied by 7.

c Mean number of hospitalizations per child ever hospitalized for asthma was calculated by dividing the number of pediatric asthma admissions by the number of children admitted for asthma.

d Analysis of readmissions within 180 days of discharge used additional records from 2001 to avoid censoring bias.

**Table 3:** Relative Risk Ratios, Pediatric Asthma Hospitalizations and Readmissions by Race/Ethnicity, Age, and Gender, New Jersey, 1994-2000

		**Relative risk ratio by age^a^**			
**Race/Ethnicity**	**Age (years)**	**Hospitalization Ratio^b^**	**Person-level Hospitalization Rate^c^**	**Mean Hospitalizations per Child Ever Hospitalized for Asthma^d^**	**Readmission Within 180 Days of Prior Discharge^e^**

**Female**					

**White**	1-4	3.0	3.1	1.0	1.1
	5-9	1.1	1.2	0.9	0.8
	10-14	--	--	--	--
**Black**	1-4	3.0	3.9	0.8	0.6
	5-9	1.3	1.6	0.8	0.7
	10-14	--	--	--	--
**Hispanic**	1-4	2.6	3.1	0.8	0.8
	5-9	1.3	1.5	0.9	1.0
	10-14	--	--	--	--

**Male**					

**White**	1-4	4.5	4.7	1.0	0.9
	5-9	1.5	1.7	0.9	0.7
	10-14	--	--	--	--
**Black**	1-4	4.1	4.7	0.9	0.8
	5-9	1.6	1.8	0.9	0.8
	10-14	--	--	--	--
**Hispanic**	1-4	4.5	4.8	0.9	1.0
	5-9	2.0	2.0	1.0	1.0
	10-14	--	--	--	--


	**Relative risk ratio by race/ethnicity^f^**			
**Race/Ethnicity**	**Age(years)**	**Hospitalization Ratio**	**Person-level Hospitalization Rate**	**Mean Hospitalizations per Child Ever Hospitalized for Asthma**	**Readmission Within 180 Days of Prior Discharge**

**Female**					

**White**	1-4	--	--	--	--
	5-9	--	--	--	--
	10-14	--	--	--	--
**Black**	1-4	4.5	4.0	1.1	1.6
	5-9	5.2	4.3	1.2	2.4
	10-14	4.4	3.2	1.4	3.0
**Hispanic**	1-4	2.1	1.8	1.2	1.7
	5-9	2.9	2.2	1.3	2.5
	10-14	2.4	1.8	1.3	2.2

**Male**					

**White**	1-4	--	--	--	--
	5-9	--	--	--	--
	10-14	--	--	--	--
**Black**	1-4	4.2	3.6	1.1	1.7
	5-9	4.8	4.0	1.2	2.1
	10-14	4.5	3.7	1.2	1.9
**Hispanic**	1-4	2.0	1.7	1.2	1.7
	5-9	2.6	2.1	1.2	2.4
	10-14	2.0	1.7	1.2	1.6

a 1-4 year and 5-9 year age groups are compared to 10-14 year age group.

b Hospital ratios are defined as the number of pediatric asthma admissions in a population subgroup, including multiple readmissions of the same patient, divided by the number of individuals in a population subgroup, as determined by the 2000 Census, multiplied by 7.

c Person-level hospitalization rates are defined as the number of pediatric asthma individuals hospitalized within a population subgroup, divided by the number of individuals in a population subgroup, as determined by the 2000 Census, multiplied by 7.

d Mean number of hospitalizations per child ever hospitalized for asthma was calculated by dividing the number of pediatric asthma admissions by the number of children admitted for asthma.

e Analysis of readmissions within 180 days of discharge used additional records from 2001 to avoid censoring bias.

f Black and Hispanic children are compared to white children.
